# Crystal structure of 7,7′-[(pyridin-2-yl)methyl­ene]bis­(5-chloro­quinolin-8-ol)

**DOI:** 10.1107/S2056989020009317

**Published:** 2020-07-14

**Authors:** Yukiyasu Kashiwagi, Koji Kubono, Toshiyuki Tamai

**Affiliations:** a Osaka Research Institute of Industrial Science and Technology, Joto-ku, Osaka, 536-8553, Japan; b Osaka Kyoiku University, Kashiwara, Osaka 582-8582, Japan

**Keywords:** crystal structure, 8-quinolinol, bridging ligand, tridentate ligand, C—H⋯π inter­actions

## Abstract

In the title compound, C_24_H_15_Cl_2_N_3_O_2_, one quinoline ring system is essentially planar and the other is slightly bent. An intra­molecular O—H⋯N hydrogen bond involving the hy­droxy group and a pyridine N atom forms an *S*(5) ring motif. In the crystal, two mol­ecules are associated into an inversion dimer with two *R*_2_^2^(7) ring motifs through inter­molecular O—H⋯N and O—H⋯O hydrogen bonds. The dimers are further linked by an inter­molecular C—H⋯O hydrogen bond and four C—H⋯π inter­actions, forming a two-dimensional network parallel to (001).

## Abstract 2

In the crystal, mol­ecules of the title compound, a potential ligand containing two 8-quinolinol and one 2-pyridine units, are linked by inter­molecular O—H⋯N and O—H⋯O quadruple hydrogen bonds, forming an inversion dimer with two 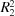
(7) ring motifs. The dimers are associated through a C—H⋯O hydrogen bond and four C—H⋯π inter­actions.

## Chemical context   

8-Quinolinol and its derivatives are well-known chelating agents in analytical chemistry and bidentate ligands to metal ions in structural chemistry. Recently, multinuclear metal complexes based on the dimeric 8-quinolinol ligand, 1,1-bis­(8-hy­droxy­quinolin-7-yl)ethane, have been investigated (Zhu *et al.*, 2012 ▸; Zhang *et al.*, 2014 ▸; Wu *et al.*, 2017 ▸; Gao *et al.*, 2018 ▸). On the other hand, Yamato *et al.* (1986 ▸, 1987 ▸) reported the aromatic-group-substituted dimeric 8-quinolinol derivatives, 1,1-bis­(8-hy­droxy­quinolin-7-yl)-1-(4-meth­oxy­phen­yl)meth­ane, 1,1-bis­(8-hy­droxy­quinolin-7-yl)-1-(furan-2-yl)methane and 1,1-bis­(8-hy­droxy­quinolin-7-yl)-1-(thio­phen-2-yl)meth­ane, to be candidates for anti­tumor agents. We are attempting to develop a 2-pyridyl group-introduced dimeric 8-quinolinol-based ligand for mono- and multi-nuclear metal complexes, and report here the crystal structure of the title compound.
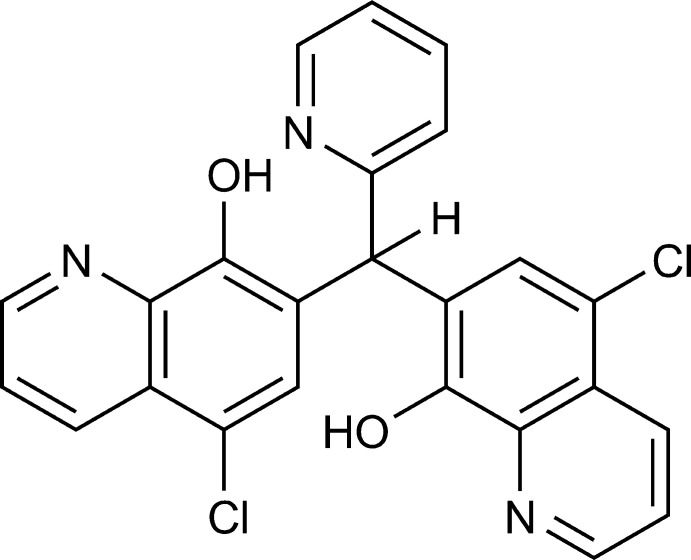


## Structural commentary   

**Figure 1:**
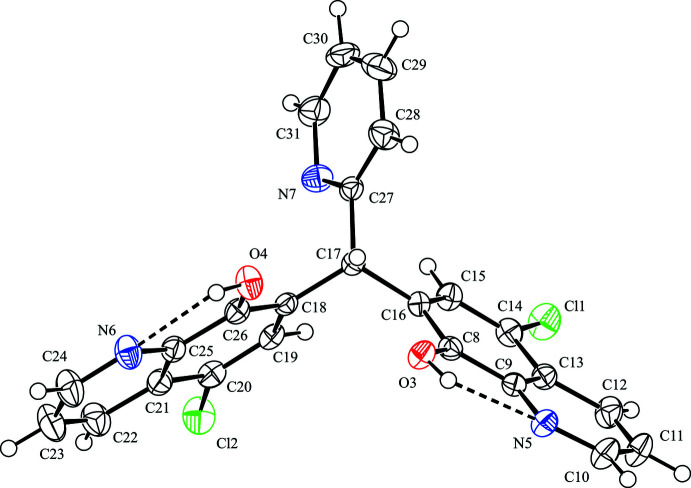
The mol­ecular structure of the title compound, with the atom labelling. Displacement ellipsoids are drawn at the 50% probability level. H atoms are represented by spheres of arbitrary radius. The intra­molecular O—H⋯N hydrogen bonds are shown as dashed lines.

The mol­ecular structure of the title compound is shown in Fig. 1 ▸. One quinoline ring system is essentially planar, the dihedral angle between the mean planes through C22–C24/N6 and C26/C18–C20 being 0.5 (2)°. The other quinoline ring system is slightly bent, the dihedral angle between the mean planes through N5/C10–C12 and C14–C16/C8 being 5.77 (18)°. There are two intra­molecular O—H⋯N hydrogen bonds involving the hydroxy groups and quinoline N atoms (O3—H3⋯N5 and O4—H4⋯N6; Table 1 ▸) generating *S*(5) ring motifs (Fig. 1 ▸). The arrangement of the 2-pyridyl and two quinoline rings is propeller-wise, which is a common arrangement for Ar_3_C-H fragments. The bond angles C16—C17—C18, C16—C17—C27, and C18—C17—C27 are 112.21 (16), 112.64 (16) and 112.94 (16)°, respectively. The torsion angles C8—C16—C17—C18, C26—C18—C17—C27, and C28—C27—C17—C16 are −88.6 (2), −101.2 (2) and −87.8 (2)°, respectively.

## Supra­molecular features   

**Figure 2:**
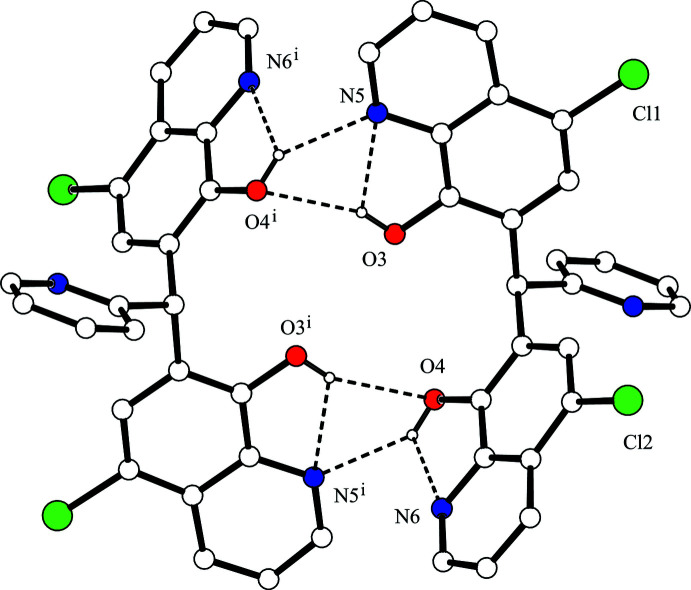
A centrosymmetric dimeric structure of the title compound. The intra- and inter­molecular hydrogen bonds are shown as dashed lines. H atoms not involved in these inter­actions have been omitted for clarity. [Symmetry code: (i) −*x* + 1, −*y* + 2, −*z* + 2.]

**Figure 3:**
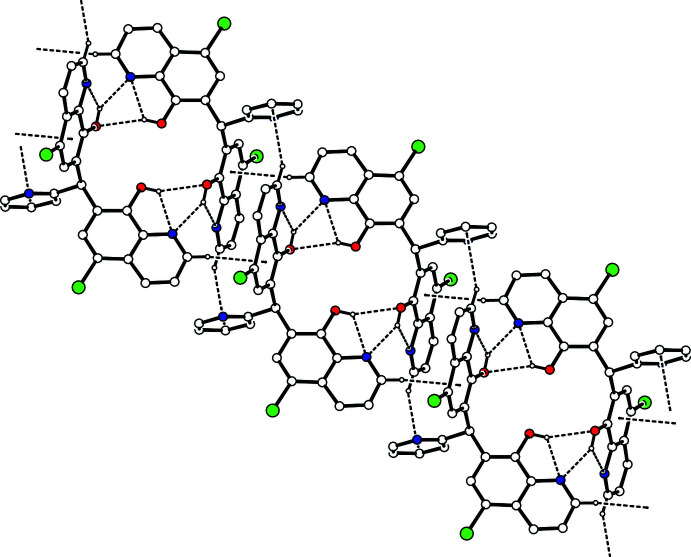
A packing diagram of the title compound, showing the ribbon structure. The C—H⋯π inter­actions between the dimers are shown as dashed lines. H atoms not involved in the inter­actions have been omitted for clarity.

**Figure 4:**
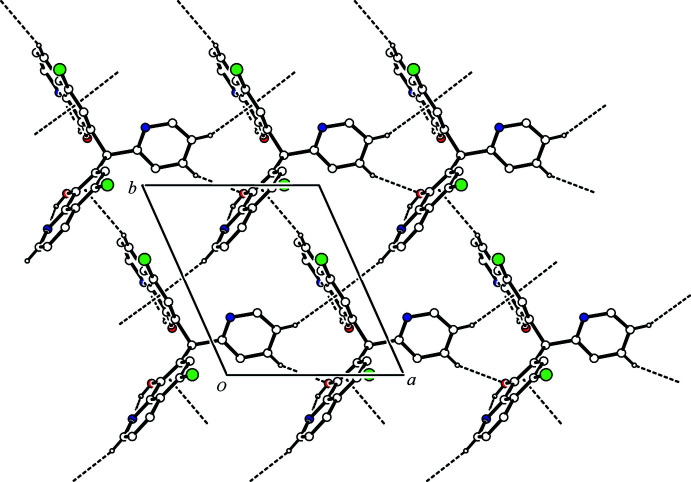
A packing diagram of the title compound viewed along the *c* axis, showing the two-dimensional network sheet structure. The C—H⋯π inter­actions and C—H⋯O hydrogen bonds are shown as dashed lines. H atoms not involved in the inter­actions have been omitted for clarity.

In the crystal, mol­ecules are linked by inter­molecular O—H⋯O and O—H⋯N hydrogen bonds [O3—H3⋯O4^i^ and O4—H4⋯N5^i^; symmetry code: (i) –*x* + 1, –*y* + 2, –*z* + 2], forming an inversion dimer with two 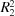
(7) ring motifs (Fig. 2 ▸ and Table 1 ▸). In contrast, the crystal structure of 1,1-bis­(8-hy­droxy­quinolin-7-yl)methane, an analogue of the title compound with the 2-pyridyl group omitted, exhibits a supra­molecular 1D-polymeric structure with inter­molecular hydrogen bonding between each 8-quinolinol unit and two other mol­ecules (CSD refcode CIBCEV; Albrecht *et al.*, 1999 ▸). The dimers of the title compound are linked by complementary C—H⋯π inter­actions [C10—H10⋯*Cg*1^iii^ and C24—H24⋯*Cg*3^v^; *Cg*1 is the centroid of the C18–C21/C25/C26 ring and *Cg*3 is the centroid of the N7/C27–C31 ring; symmetry codes: (iii) *x* + 1, *y* + 1, *z*; (v) –*x*, –*y* + 1, –*z* + 2], forming a ribbon structure along [110] (Fig. 3 ▸). Considered separately, the 1D-chain structure propagates along the *a*-axis direction through a C—H⋯O hydrogen bond [C29—H29⋯O3^ii^; symmetry code: (ii) *x* – 1, *y*, *z*] and a C—H⋯π inter­action [C30—H30⋯*Cg*1^ii^; *Cg*1 is the centroid of the C18–C21/C25/C26 ring]. The chains are linked by two C—H⋯π inter­actions [C10—H10⋯*Cg*1^iii^ and C22—H22⋯*Cg*2^iv^; *Cg*2 is the centroid of the C8/C9/C13–C16 ring; symmetry code: (iv) *x*, *y* – 1, *z*], generating a two-dimensional network parallel to (001) (Fig. 4 ▸).

## Database survey   

A search of the Cambridge Structural Database (CSD, Version 5.41, update of March 2020; Groom *et al.*, 2016 ▸) for compounds containing the bis­(phenol-2-yl)methane skeleton gave 9360 hits, and for those containing the 8-quinolinol skeleton gave 3200 hits. A search for the fragment of 1,1-bis­(8-hy­droxy­quinolin-7-yl)methane gave 23 hits (21 compounds), which included only one organic compound, 1,1-bis­(8-hy­droxy­quinolin-7-yl)methane (CIBCEV; Albrecht *et al.*, 1999 ▸), and 20 metal complexes with 1,1-bis­(8-hy­droxy­quinolin-7-yl)ethane as bridging ligands. The 20 metal complexes include two dinuclear complexes, Zn_2_ (Wu *et al.*, 2017 ▸; Gao *et al.*, 2018 ▸) and Cd_2_ (Gao *et al.*, 2018 ▸), one homo-trinuclear La_3_ complex (Wu *et al.*, 2017 ▸), 16 hetero-trinuclear complexes, Co_2_Sm, Ni_2_Sm, Zn_2_Sm, Co_2_Eu, Ni_2_Eu, Zn_2_Eu, Cd_2_Eu, Co_2_Gd, Ni_2_Gd, Cd_2_Gd, Co_2_Tb, Ni_2_Tb, Zn_2_Tb, Fe_2_Dy, Co_2_Dy, Cd_2_Dy (Zhu *et al.*, 2012 ▸) and one hexa­nuclear Na_2_Co_4_ complex (Zhang *et al.*, 2014 ▸). The crystal structure of 1,1-bis­(8-hy­droxy­quinolin-7-yl)ethane itself has not been reported.

## Synthesis and crystallization   

The title compound was prepared by a modification of the reported K_2_CO_3_-catalysed synthetic method for 1,1-bis­(5-chloro-8-hy­droxy­quinolin-7-yl)methane (Ozawa & Shibuya, 1963*a* ▸,*b* ▸). 5-Chloro-8-hy­droxy­quinoline (898 mg, 5.0 mmol), 2-pyridine­carboxaldehyde (321 mg, 3.0 mmol), K_2_CO_3_ (100 mg, 0.72 mmol) and ethanol (6 mL) were placed in a 15 mL capped pressure tube. It was heated at 353 K for 96 h. The generated pale-white precipitate was filtered to give a pale-white solid (806 mg, 1.80 mmol; yield 72%). Single crystals of title compound suitable for X-ray diffraction were grown by slow evaporation of a solution in CHCl_3_/*n*-hexane (2:1, *v*/*v*) at ambient temperature. ^1^H NMR (CDCl_3_, 600 MHz) *δ* = 6.63 (*s*, 1H), 7.21 (*ddd*, 1H, *J* = 7.8, 4.8, 1.8 Hz), 7.33 (*d*, 1H, *J* = 7.8 Hz), 7.52 (*s*, 2H), 7.52 (*dd*, 2H, *J* = 8.4, 4.2 Hz), 7.67 (*td*, 1H, *J* = 7.8, 1.8 Hz), 8.48 (*dd*, 2H, *J* = 8.4, 1.2 Hz), 8.64 (*d*, 1H, *J* = 4.8 Hz), 8.81 (*dd*, 2H, *J* = 4.2, 1.2 Hz), 8.84 (*br*, 2H).

## Refinement   

Crystal data, data collection and structure refinement details are summarized in Table 2 ▸. Hydroxy H atoms were located in a difference-Fourier map and freely refined. C-bound H atoms were placed in geometrically calculated positions (C—H = 0.95–1.00 Å) and refined as part of a riding model with *U*_iso_(H) = 1.2*U*_eq_ (C). One outlier (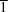
11) was omitted from the refinement.

## Supplementary Material

Crystal structure: contains datablock(s) global, I. DOI: 10.1107/S2056989020009317/is5545sup1.cif

Structure factors: contains datablock(s) I. DOI: 10.1107/S2056989020009317/is5545Isup2.hkl

Supporting information file. DOI: 10.1107/S2056989020009317/is5545Isup3.cml

CCDC reference: 2014831

Additional supporting information:  crystallographic information; 3D view; checkCIF report

## Figures and Tables

**Table 1:** Hydrogen-bond geometry (Å, °) *Cg*1, *Cg*2 and *Cg*3 are the centroids of the C18–C21/C25/C26, C8/C9/C13–C16 and N7/C27–C31 rings, respectively.

*D*—H⋯*A*	*D*—H	H⋯*A*	*D*⋯*A*	*D*—H⋯*A*
O3—H3⋯N5	0.82 (2)	2.23 (3)	2.727 (2)	119 (2)
O3—H3⋯O4^i^	0.83 (3)	2.26 (3)	3.044 (2)	159 (2)
O4—H4⋯N5^i^	0.84 (4)	2.16 (3)	2.878 (3)	144 (2)
O4—H4⋯N6	0.84 (4)	2.20 (4)	2.674 (3)	115 (3)
C29—H29⋯O3^ii^	0.95	2.53	3.377 (3)	148
C10—H10⋯*Cg*1^iii^	0.95	2.75	3.659 (3)	161
C22—H22⋯*Cg*2^iv^	0.95	2.92	3.667 (3)	136
C24—H24⋯*Cg*3^v^	0.95	2.65	3.528 (3)	153
C30—H30⋯*Cg*1^ii^	0.95	2.73	3.562 (3)	147

Symmetry codes: (i) 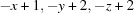; (ii) 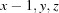; (iii) 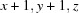; (iv) 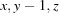; (v) 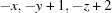.

**Table 2:** Experimental details

Crystal data	
Chemical formula	C_24_H_15_Cl_2_N_3_O_2_
*M*_r_	448.31
Crystal system, space group	Triclinic, *P* 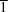
Temperature (K)	173
*a*, *b*, *c* (Å)	8.7077 (8), 10.4281 (10), 12.1329 (11)
α, β, γ (°)	101.111 (7), 92.087 (7), 113.161 (8)
*V* (Å^3^)	986.23 (17)
*Z*	2
Radiation type	Mo *K*α
μ (mm^−1^)	0.36
Crystal size (mm)	0.40 × 0.15 × 0.10

Data collection	
Diffractometer	Rigaku R-AXIS RAPID
Absorption correction	Multi-scan (*ABSCOR*; Higashi, 1995 ▸)
*T*_min_, *T*_max_	0.710, 0.965
No. of measured, independent and observed [*F* ^2^ > 2.0σ(*F* ^2^)] reflections	9484, 4475, 3536
*R*_int_	0.027
(sin θ/λ)_max_ (Å^−1^)	0.649

Refinement	
*R*[*F* ^2^ > 2σ(*F* ^2^)], *wR*(*F* ^2^), *S*	0.050, 0.119, 1.07
No. of reflections	4475
No. of parameters	288
H-atom treatment	H atoms treated by a mixture of independent and constrained refinement
Δρ_max_, Δρ_min_ (e Å^−3^)	0.43, −0.22

Computer programs: *RAPID-AUTO* (Rigaku, 2006 ▸), *SIR92* (Altomare *et al.*, 1993 ▸), *SHELXL2014/7* (Sheldrick, 2015 ▸), *PLATON* (Spek, 2020 ▸) and *CrystalStructure* (Rigaku, 2016 ▸).

## supplementary crystallographic information

### Crystal data

**Table:**

C_24_H_15_Cl_2_N_3_O_2_	*Z* = 2
*M**_r_* = 448.31	*F*(000) = 460.00
Triclinic, *P*1	*D*_x_ = 1.510 Mg m^−^^3^
*a* = 8.7077 (8) Å	Mo *K*α radiation, λ = 0.71075 Å
*b* = 10.4281 (10) Å	Cell parameters from 7587 reflections
*c* = 12.1329 (11) Å	θ = 3.0–27.5°
α = 101.111 (7)°	µ = 0.36 mm^−^^1^
β = 92.087 (7)°	*T* = 173 K
γ = 113.161 (8)°	Chunk, colorless
*V* = 986.23 (17) Å^3^	0.40 × 0.15 × 0.10 mm

### Data collection

**Table:**

Rigaku R-AXIS RAPID diffractometer	3536 reflections with *F*^2^ > 2.0σ(*F*^2^)
Detector resolution: 10.000 pixels mm^-1^	*R*_int_ = 0.027
ω scans	θ_max_ = 27.5°, θ_min_ = 3.1°
Absorption correction: multi-scan (ABSCOR; Higashi, 1995)	*h* = −11→11
*T*_min_ = 0.710, *T*_max_ = 0.965	*k* = −13→13
9484 measured reflections	*l* = −15→15
4475 independent reflections	

### Refinement

**Table:**

Refinement on *F*^2^	Secondary atom site location: difference Fourier map
*R*[*F*^2^ > 2σ(*F*^2^)] = 0.050	Hydrogen site location: inferred from neighbouring sites
*wR*(*F*^2^) = 0.119	H atoms treated by a mixture of independent and constrained refinement
*S* = 1.07	*w* = 1/[σ^2^(*F*_o_^2^) + (0.0532*P*)^2^ + 0.572*P*] where *P* = (*F*_o_^2^ + 2*F*_c_^2^)/3
4475 reflections	(Δ/σ)_max_ < 0.001
288 parameters	Δρ_max_ = 0.43 e Å^−^^3^
0 restraints	Δρ_min_ = −0.22 e Å^−^^3^
Primary atom site location: structure-invariant direct methods	

### Special details

**Table:**

Geometry. All esds (except the esd in the dihedral angle between two l.s. planes) are estimated using the full covariance matrix. The cell esds are taken into account individually in the estimation of esds in distances, angles and torsion angles; correlations between esds in cell parameters are only used when they are defined by crystal symmetry. An approximate (isotropic) treatment of cell esds is used for estimating esds involving l.s. planes.
Refinement. Refinement was performed using all reflections. The weighted R-factor (wR) and goodness of fit (S) are based on F^2^. R-factor (gt) are based on F. The threshold expression of F^2^ > 2.0 sigma(F^2^) is used only for calculating R-factor (gt).

### Fractional atomic coordinates and isotropic or equivalent isotropic displacement parameters (Å^2^)

**Table:**

	*x*	*y*	*z*	*U*_iso_*/*U*_eq_	
Cl1	0.19486 (7)	0.99797 (7)	0.42458 (5)	0.03826 (17)	
Cl2	0.17365 (8)	0.39091 (6)	0.56639 (5)	0.03966 (17)	
O3	0.44767 (19)	1.04439 (17)	0.89332 (12)	0.0268 (3)	
O4	0.1923 (2)	0.75295 (16)	1.00827 (13)	0.0305 (3)	
N5	0.6459 (2)	1.23152 (19)	0.77548 (15)	0.0269 (4)	
N6	0.2352 (3)	0.5120 (2)	1.00448 (15)	0.0328 (4)	
N7	−0.1639 (2)	0.6963 (2)	0.71721 (16)	0.0306 (4)	
C8	0.3918 (2)	1.0343 (2)	0.78420 (16)	0.0216 (4)	
C9	0.4946 (2)	1.1297 (2)	0.72083 (16)	0.0220 (4)	
C10	0.7377 (3)	1.3256 (2)	0.7197 (2)	0.0340 (5)	
H10	0.8414	1.3988	0.7577	0.041*	
C11	0.6909 (3)	1.3234 (3)	0.6073 (2)	0.0367 (5)	
H11	0.7630	1.3927	0.5706	0.044*	
C12	0.5418 (3)	1.2218 (2)	0.55110 (19)	0.0322 (5)	
H12	0.5093	1.2188	0.4747	0.039*	
C13	0.4351 (3)	1.1202 (2)	0.60802 (17)	0.0246 (4)	
C14	0.2728 (3)	1.0141 (2)	0.56284 (17)	0.0260 (4)	
C15	0.1743 (3)	0.9261 (2)	0.62662 (17)	0.0257 (4)	
H15	0.0646	0.8576	0.5945	0.031*	
C16	0.2326 (2)	0.9353 (2)	0.73891 (16)	0.0217 (4)	
C17	0.1239 (2)	0.8422 (2)	0.81349 (17)	0.0218 (4)	
H17	0.1639	0.8989	0.8932	0.026*	
C18	0.1510 (2)	0.7059 (2)	0.80759 (16)	0.0207 (4)	
C19	0.1464 (2)	0.6152 (2)	0.70280 (17)	0.0235 (4)	
H19	0.1239	0.6396	0.6343	0.028*	
C20	0.1734 (3)	0.4941 (2)	0.69780 (17)	0.0261 (4)	
C21	0.2042 (3)	0.4507 (2)	0.79740 (18)	0.0263 (4)	
C22	0.2328 (3)	0.3278 (3)	0.8022 (2)	0.0369 (5)	
H22	0.2312	0.2638	0.7344	0.044*	
C23	0.2627 (4)	0.3015 (3)	0.9047 (2)	0.0436 (6)	
H23	0.2833	0.2196	0.9091	0.052*	
C24	0.2627 (3)	0.3965 (3)	1.0039 (2)	0.0401 (6)	
H24	0.2839	0.3763	1.0746	0.048*	
C25	0.2070 (2)	0.5402 (2)	0.90232 (17)	0.0243 (4)	
C26	0.1822 (2)	0.6680 (2)	0.90558 (17)	0.0231 (4)	
C27	−0.0611 (2)	0.8146 (2)	0.79228 (16)	0.0227 (4)	
C28	−0.1171 (3)	0.9116 (2)	0.8521 (2)	0.0342 (5)	
H28	−0.0398	0.9966	0.9023	0.041*	
C29	−0.2853 (3)	0.8838 (3)	0.8384 (2)	0.0414 (6)	
H29	−0.3263	0.9482	0.8799	0.050*	
C30	−0.3934 (3)	0.7608 (3)	0.7633 (2)	0.0390 (6)	
H30	−0.5106	0.7378	0.7530	0.047*	
C31	−0.3284 (3)	0.6725 (3)	0.7037 (2)	0.0356 (5)	
H31	−0.4031	0.5899	0.6498	0.043*	
H3	0.541 (3)	1.112 (3)	0.909 (2)	0.030 (7)*	
H4	0.214 (4)	0.718 (3)	1.060 (3)	0.052 (9)*	

### Atomic displacement parameters (Å^2^)

**Table:**

	*U*^11^	*U*^22^	*U*^33^	*U*^12^	*U*^13^	*U*^23^
Cl1	0.0417 (3)	0.0466 (3)	0.0227 (3)	0.0125 (3)	−0.0028 (2)	0.0125 (2)
Cl2	0.0575 (4)	0.0339 (3)	0.0246 (3)	0.0183 (3)	0.0078 (2)	0.0000 (2)
O3	0.0222 (8)	0.0304 (8)	0.0230 (7)	0.0050 (7)	−0.0010 (6)	0.0086 (6)
O4	0.0446 (9)	0.0283 (8)	0.0195 (7)	0.0161 (7)	0.0020 (6)	0.0051 (6)
N5	0.0237 (9)	0.0269 (9)	0.0270 (9)	0.0066 (7)	0.0032 (7)	0.0071 (7)
N6	0.0452 (11)	0.0309 (10)	0.0254 (9)	0.0176 (9)	0.0036 (8)	0.0088 (8)
N7	0.0249 (9)	0.0330 (10)	0.0300 (10)	0.0097 (8)	0.0007 (7)	0.0033 (8)
C8	0.0229 (10)	0.0233 (9)	0.0208 (9)	0.0112 (8)	0.0030 (7)	0.0058 (8)
C9	0.0225 (9)	0.0219 (9)	0.0226 (10)	0.0102 (8)	0.0043 (7)	0.0049 (8)
C10	0.0273 (11)	0.0328 (12)	0.0346 (12)	0.0032 (10)	0.0044 (9)	0.0107 (10)
C11	0.0352 (12)	0.0351 (12)	0.0362 (13)	0.0058 (10)	0.0110 (10)	0.0176 (10)
C12	0.0395 (13)	0.0355 (12)	0.0244 (11)	0.0150 (10)	0.0086 (9)	0.0130 (9)
C13	0.0269 (10)	0.0250 (10)	0.0238 (10)	0.0117 (9)	0.0059 (8)	0.0070 (8)
C14	0.0312 (11)	0.0284 (10)	0.0196 (10)	0.0136 (9)	0.0007 (8)	0.0054 (8)
C15	0.0247 (10)	0.0246 (10)	0.0245 (10)	0.0072 (8)	−0.0002 (8)	0.0046 (8)
C16	0.0229 (10)	0.0206 (9)	0.0232 (10)	0.0091 (8)	0.0036 (7)	0.0075 (8)
C17	0.0198 (9)	0.0225 (9)	0.0215 (9)	0.0063 (8)	0.0020 (7)	0.0063 (7)
C18	0.0164 (9)	0.0209 (9)	0.0231 (10)	0.0051 (8)	0.0028 (7)	0.0064 (8)
C19	0.0203 (9)	0.0259 (10)	0.0208 (9)	0.0045 (8)	0.0034 (7)	0.0077 (8)
C20	0.0256 (10)	0.0268 (10)	0.0208 (10)	0.0071 (9)	0.0040 (8)	0.0018 (8)
C21	0.0254 (10)	0.0245 (10)	0.0280 (11)	0.0084 (8)	0.0053 (8)	0.0067 (8)
C22	0.0492 (14)	0.0313 (11)	0.0344 (12)	0.0212 (11)	0.0090 (10)	0.0058 (10)
C23	0.0650 (17)	0.0361 (13)	0.0433 (14)	0.0318 (13)	0.0115 (12)	0.0145 (11)
C24	0.0578 (16)	0.0375 (13)	0.0341 (13)	0.0254 (12)	0.0057 (11)	0.0154 (11)
C25	0.0235 (10)	0.0244 (10)	0.0249 (10)	0.0084 (8)	0.0030 (8)	0.0083 (8)
C26	0.0222 (10)	0.0223 (9)	0.0214 (9)	0.0061 (8)	0.0027 (7)	0.0036 (8)
C27	0.0210 (9)	0.0251 (10)	0.0229 (10)	0.0086 (8)	0.0035 (7)	0.0095 (8)
C28	0.0341 (12)	0.0300 (11)	0.0366 (13)	0.0137 (10)	0.0016 (9)	0.0025 (9)
C29	0.0428 (14)	0.0507 (15)	0.0446 (14)	0.0324 (12)	0.0123 (11)	0.0122 (12)
C30	0.0205 (11)	0.0566 (15)	0.0473 (14)	0.0167 (11)	0.0079 (9)	0.0257 (12)
C31	0.0244 (11)	0.0373 (12)	0.0388 (13)	0.0067 (10)	−0.0035 (9)	0.0086 (10)

### Geometric parameters (Å, º)

**Table:**

Cl1—C14	1.738 (2)	C16—C17	1.528 (3)
Cl2—C20	1.744 (2)	C17—C18	1.519 (3)
O3—C8	1.364 (2)	C17—C27	1.524 (3)
O3—H3	0.82 (3)	C17—H17	1.0000
O4—C26	1.360 (2)	C18—C26	1.373 (3)
O4—H4	0.84 (3)	C18—C19	1.420 (3)
N5—C10	1.319 (3)	C19—C20	1.364 (3)
N5—C9	1.366 (3)	C19—H19	0.9500
N6—C24	1.316 (3)	C20—C21	1.421 (3)
N6—C25	1.363 (3)	C21—C22	1.410 (3)
N7—C27	1.338 (3)	C21—C25	1.419 (3)
N7—C31	1.352 (3)	C22—C23	1.362 (3)
C8—C16	1.374 (3)	C22—H22	0.9500
C8—C9	1.423 (3)	C23—C24	1.403 (4)
C9—C13	1.417 (3)	C23—H23	0.9500
C10—C11	1.402 (3)	C24—H24	0.9500
C10—H10	0.9500	C25—C26	1.425 (3)
C11—C12	1.360 (3)	C27—C28	1.385 (3)
C11—H11	0.9500	C28—C29	1.373 (3)
C12—C13	1.420 (3)	C28—H28	0.9500
C12—H12	0.9500	C29—C30	1.378 (4)
C13—C14	1.415 (3)	C29—H29	0.9500
C14—C15	1.371 (3)	C30—C31	1.369 (4)
C15—C16	1.409 (3)	C30—H30	0.9500
C15—H15	0.9500	C31—H31	0.9500
			
C8—O3—H3	106.4 (17)	C19—C18—C17	121.94 (17)
C26—O4—H4	110 (2)	C20—C19—C18	121.80 (18)
C10—N5—C9	117.43 (18)	C20—C19—H19	119.1
C24—N6—C25	117.4 (2)	C18—C19—H19	119.1
C27—N7—C31	117.0 (2)	C19—C20—C21	121.58 (19)
O3—C8—C16	118.84 (17)	C19—C20—Cl2	119.47 (16)
O3—C8—C9	120.08 (17)	C21—C20—Cl2	118.94 (16)
C16—C8—C9	121.02 (18)	C22—C21—C25	116.8 (2)
N5—C9—C13	123.10 (17)	C22—C21—C20	126.4 (2)
N5—C9—C8	116.96 (17)	C25—C21—C20	116.81 (19)
C13—C9—C8	119.88 (17)	C23—C22—C21	119.5 (2)
N5—C10—C11	123.6 (2)	C23—C22—H22	120.3
N5—C10—H10	118.2	C21—C22—H22	120.3
C11—C10—H10	118.2	C22—C23—C24	119.4 (2)
C12—C11—C10	119.7 (2)	C22—C23—H23	120.3
C12—C11—H11	120.1	C24—C23—H23	120.3
C10—C11—H11	120.1	N6—C24—C23	123.7 (2)
C11—C12—C13	119.2 (2)	N6—C24—H24	118.1
C11—C12—H12	120.4	C23—C24—H24	118.1
C13—C12—H12	120.4	N6—C25—C21	123.20 (19)
C14—C13—C9	117.62 (17)	N6—C25—C26	116.15 (19)
C14—C13—C12	125.45 (19)	C21—C25—C26	120.64 (18)
C9—C13—C12	116.90 (19)	O4—C26—C18	120.59 (18)
C15—C14—C13	121.40 (19)	O4—C26—C25	118.42 (18)
C15—C14—Cl1	119.35 (16)	C18—C26—C25	120.99 (18)
C13—C14—Cl1	119.23 (15)	N7—C27—C28	122.48 (19)
C14—C15—C16	121.20 (18)	N7—C27—C17	118.58 (18)
C14—C15—H15	119.4	C28—C27—C17	118.94 (18)
C16—C15—H15	119.4	C29—C28—C27	119.5 (2)
C8—C16—C15	118.83 (17)	C29—C28—H28	120.3
C8—C16—C17	118.67 (17)	C27—C28—H28	120.3
C15—C16—C17	122.48 (17)	C28—C29—C30	118.7 (2)
C18—C17—C27	112.94 (16)	C28—C29—H29	120.7
C18—C17—C16	112.21 (16)	C30—C29—H29	120.7
C27—C17—C16	112.64 (16)	C31—C30—C29	118.7 (2)
C18—C17—H17	106.1	C31—C30—H30	120.6
C27—C17—H17	106.1	C29—C30—H30	120.6
C16—C17—H17	106.1	N7—C31—C30	123.6 (2)
C26—C18—C19	118.16 (18)	N7—C31—H31	118.2
C26—C18—C17	119.90 (17)	C30—C31—H31	118.2
			
C10—N5—C9—C13	1.1 (3)	C18—C19—C20—C21	1.3 (3)
C10—N5—C9—C8	−176.2 (2)	C18—C19—C20—Cl2	−177.68 (15)
O3—C8—C9—N5	−1.8 (3)	C19—C20—C21—C22	179.7 (2)
C16—C8—C9—N5	175.27 (19)	Cl2—C20—C21—C22	−1.3 (3)
O3—C8—C9—C13	−179.15 (18)	C19—C20—C21—C25	−0.6 (3)
C16—C8—C9—C13	−2.0 (3)	Cl2—C20—C21—C25	178.43 (15)
C9—N5—C10—C11	−2.1 (3)	C25—C21—C22—C23	−0.6 (3)
N5—C10—C11—C12	1.3 (4)	C20—C21—C22—C23	179.2 (2)
C10—C11—C12—C13	0.6 (4)	C21—C22—C23—C24	0.7 (4)
N5—C9—C13—C14	−177.04 (19)	C25—N6—C24—C23	−0.7 (4)
C8—C9—C13—C14	0.1 (3)	C22—C23—C24—N6	0.0 (4)
N5—C9—C13—C12	0.7 (3)	C24—N6—C25—C21	0.7 (3)
C8—C9—C13—C12	177.9 (2)	C24—N6—C25—C26	−178.3 (2)
C11—C12—C13—C14	176.0 (2)	C22—C21—C25—N6	−0.1 (3)
C11—C12—C13—C9	−1.5 (3)	C20—C21—C25—N6	−179.89 (19)
C9—C13—C14—C15	1.7 (3)	C22—C21—C25—C26	178.89 (19)
C12—C13—C14—C15	−175.8 (2)	C20—C21—C25—C26	−0.9 (3)
C9—C13—C14—Cl1	−179.77 (16)	C19—C18—C26—O4	178.27 (17)
C12—C13—C14—Cl1	2.7 (3)	C17—C18—C26—O4	−1.2 (3)
C13—C14—C15—C16	−1.7 (3)	C19—C18—C26—C25	−0.9 (3)
Cl1—C14—C15—C16	179.83 (16)	C17—C18—C26—C25	179.60 (17)
O3—C8—C16—C15	179.29 (18)	N6—C25—C26—O4	1.5 (3)
C9—C8—C16—C15	2.1 (3)	C21—C25—C26—O4	−177.55 (18)
O3—C8—C16—C17	1.0 (3)	N6—C25—C26—C18	−179.29 (18)
C9—C8—C16—C17	−176.17 (18)	C21—C25—C26—C18	1.6 (3)
C14—C15—C16—C8	−0.3 (3)	C31—N7—C27—C28	−1.7 (3)
C14—C15—C16—C17	177.9 (2)	C31—N7—C27—C17	177.57 (18)
C8—C16—C17—C18	−88.6 (2)	C18—C17—C27—N7	−35.5 (2)
C15—C16—C17—C18	93.1 (2)	C16—C17—C27—N7	92.9 (2)
C8—C16—C17—C27	142.58 (19)	C18—C17—C27—C28	143.83 (19)
C15—C16—C17—C27	−35.7 (3)	C16—C17—C27—C28	−87.8 (2)
C27—C17—C18—C26	−101.2 (2)	N7—C27—C28—C29	2.8 (3)
C16—C17—C18—C26	130.17 (19)	C17—C27—C28—C29	−176.4 (2)
C27—C17—C18—C19	79.3 (2)	C27—C28—C29—C30	−1.3 (4)
C16—C17—C18—C19	−49.3 (2)	C28—C29—C30—C31	−1.2 (4)
C26—C18—C19—C20	−0.6 (3)	C27—N7—C31—C30	−1.0 (3)
C17—C18—C19—C20	178.93 (18)	C29—C30—C31—N7	2.5 (4)

### Hydrogen-bond geometry (Å, º)

*Cg*1, *Cg*2 and *Cg*3 are the centroids of the C18–C21/C25/C26, C8/C9/C13–C16 and N7/C27–C31 rings, respectively.

**Table:**

*D*—H···*A*	*D*—H	H···*A*	*D*···*A*	*D*—H···*A*
O3—H3···N5	0.82 (2)	2.23 (3)	2.727 (2)	119 (2)
O3—H3···O4^i^	0.83 (3)	2.26 (3)	3.044 (2)	159 (2)
O4—H4···N5^i^	0.84 (4)	2.16 (3)	2.878 (3)	144 (2)
O4—H4···N6	0.84 (4)	2.20 (4)	2.674 (3)	115 (3)
C29—H29···O3^ii^	0.95	2.53	3.377 (3)	148
C10—H10···*Cg*1^iii^	0.95	2.75	3.659 (3)	161
C22—H22···*Cg*2^iv^	0.95	2.92	3.667 (3)	136
C24—H24···*Cg*3^v^	0.95	2.65	3.528 (3)	153
C30—H30···*Cg*1^ii^	0.95	2.73	3.562 (3)	147

Symmetry codes: (i) −*x*+1, −*y*+2, −*z*+2; (ii) *x*−1, *y*, *z*; (iii) *x*+1, *y*+1, *z*; (iv) *x*, *y*−1, *z*; (v) −*x*, −*y*+1, −*z*+2.
